# Interpretable high-order knowledge graph neural network for predicting synthetic lethality in human cancers

**DOI:** 10.1093/bib/bbaf142

**Published:** 2025-04-07

**Authors:** Xuexin Chen, Ruichu Cai, Zhengting Huang, Zijian Li, Jie Zheng, Min Wu

**Affiliations:** School of Computer Science, Guangdong University of Technology, No. 100 Waihuan Xi Road, Panyu, Guangdong, Guangzhou, 510006, China; School of Computer Science, Guangdong University of Technology, No. 100 Waihuan Xi Road, Panyu, Guangdong, Guangzhou, 510006, China; Pazhou Laboratory (Huangpu), No. 248 Pazhou Qiaotou Street, Haizhu, Guangdong Province, Guangzhou, 510335, China; School of Computer Science, Guangdong University of Technology, No. 100 Waihuan Xi Road, Panyu, Guangdong, Guangzhou, 510006, China; Machine Learning Department, Mohamed bin Zayed University of Artificial Intelligence, Masdar, Abu Dhabi, United Arab Emirates; School of Information Science and Technology, ShanghaiTech University, No. 393 Huaxia Middle Road, Pudong, Shanghai, 201210, China; School of Information Science and Technology, Shanghai Engineering Research Center of Intelligent Vision and Imaging, ShanghaiTech University, No. 393 Huaxia Middle Road, Pudong, Shanghai, 201210, China; Institute for Infocomm Research (I^2^R), A*STAR, No. 2 Fusionopolis Way, Queenstown Planning, Singapore 138632, Singapore

**Keywords:** synthetic lethality, machine learning explainability, graph neural network, information bottleneck

## Abstract

Synthetic lethality (SL) is a promising gene interaction for cancer therapy. Recent SL prediction methods integrate knowledge graphs (KGs) into graph neural networks (GNNs) and employ attention mechanisms to extract local subgraphs as explanations for target gene pairs. However, attention mechanisms often lack fidelity, typically generate a single explanation per gene pair, and fail to ensure trustworthy high-order structures in their explanations. To overcome these limitations, we propose Diverse Graph Information Bottleneck for Synthetic Lethality (DGIB4SL), a KG-based GNN that generates multiple faithful explanations for the same gene pair and effectively encodes high-order structures. Specifically, we introduce a novel DGIB objective, integrating a determinant point process constraint into the standard information bottleneck objective, and employ 13 motif-based adjacency matrices to capture high-order structures in gene representations. Experimental results show that DGIB4SL outperforms state-of-the-art baselines and provides multiple explanations for SL prediction, revealing diverse biological mechanisms underlying SL inference.

## Introduction

Synthetic lethality (SL) is a promising type of genetic interaction where the co-occurrence of two (or more) genetic events leads to cell death, while the occurrence of either event is compatible with cell viability. SL has become a cornerstone of anticancer drug research, by targeting a gene that is nonessential in normal cells but synthetic lethal with a gene with cancer-specific alterations, which would enable the selective killing of cancer cells without harming normal cells. For example, AZD1775, a WEE1 Inhibitor, is based on the SL interaction between WEE1 and p53 mutations [1]. Despite extensive research on SL through high-throughput wet-lab screening methods, these methods often face various challenges, such as high costs and inconsistencies across platforms. Thus, predicting SL using computational models becomes highly complementary to wet-lab approaches.

SL prediction approaches can be broadly categorized into statistical inference methods, network-based methods, and supervised machine learning (ML) methods. Among these, graph neural networks (GNNs) are currently the most popular model, largely owing to their ability to model complex gene interactions [2]. Although many SL gene pairs have been identified, few of them have been applied to cancer treatment, as understanding the underlying biological mechanisms remains a critical challenge. Unfortunately, most GNNs lack the capability to explain SL mechanisms. To address this, methods incorporating attention mechanisms and knowledge graphs (KGs), a heterogeneous graph containing biological entities and their relationships, have emerged [2–5]. These approaches enable the identification of crucial edges or semantic features in KGs while predicting SL interaction.

**Figure 1:**
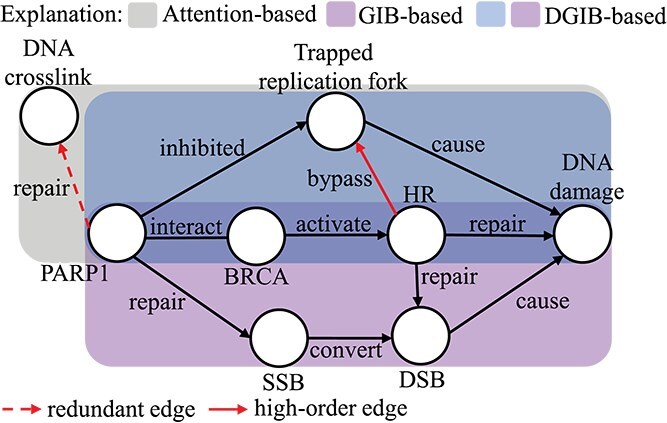
Toy example of a KG with self-loops integrating biological context and relevant mechanisms between the given gene pair BRCA1 and PARP1. The purple and blue subgraphs illustrate mechanisms where either the conversion of SSBs to DSBs or the blockage of replication forks leads to DNA damage in the absence of HR. The gray subgraph represents the predicted core subgraph of an attention-based method. A GIB-based method identifies only one correct subgraph, while our DGIB4SL can find all correct subgraphs (purple and blue). The self-loops are not depicted for brevity.

Although KG-based methods with attention mechanisms improve the interpretability of SL predictions, they still face three major challenges. First, explanations based on the attention mechanisms often lack reliability, since they tend to assign higher weights to frequent edges and produce unstable explanations across independent runs of the same model [6–10]. As illustrated by the examples in Fig. 1, the gray subgraph, predicted by attention-based methods, includes a red dashed edge labeled “repair.” This edge, irrelevant to the SL mechanism, is assigned higher importance due to its frequent occurrence in the KG. Second, existing KG-based methods generate only a single core subgraph to explain predictions for a given gene pair, even though multiple subgraphs may provide valid explanations [11]. As illustrated in Fig. 1, the purple subgraph highlights a mechanism where single-strand break (SSB) converts to double-strand break (DSB), while the blue subgraph represents replication fork blocking. Both subgraphs explain the SL interaction between PARP1 and BRCA [11]. Third, the high-order structures contained in the explanations generated by KG-based methods are often untrustworthy, since the key step of these self-explainable methods, learning gene representation for prediction, cannot capture the information of the interactions between the neighbors (high-order), although the information between a gene and its neighbors can be effectively captured (low-order). For instance, as shown in Fig. 1, the “DAN damage” node representation produced by KG-based methods remains unchanged, regardless of the high-order edge “HR $\xrightarrow{\text{bypass}}$ Trapped replication fork.” We thus ask: for a gene pair, how to find multiple rather than one faithful core subgraphs and encode their high-order graph information for prediction?

Our main contribution lies in addressing this question by proposing the Diverse Graph Information Bottleneck for Synthetic Lethality (DGIB4SL), an interpretable GNN model for SL prediction on KGs, hinging on the motif-based GNN encoder and our proposed DGIB objective. First, to alleviate instability and the bias toward frequent edges in attention weights, unlike the cross-entropy loss commonly used in attention-based methods, DGIB4SL employs the GIB principle [12], widely applied in interpretable GNNs [13, 14], to define a core subgraph from the neighborhood of a gene pair. GIB provides a principled objective function for graph representation learning (GRL), determining which data aspects to preserve and discard [15]. However, the standard GIB objective identifies only a single core subgraph for each gene pair. To capture all relevant core subgraphs from the enclosing graph, such as the purple and blue subgraphs in Fig. 1, we propose the novel Diverse GIB (DGIB) objective function, which incorporates a determinant point process (DPP) [16] constraint into GIB. DPP quantifies diversity by measuring differences between core subgraphs through the determinant of the inner product of their subgraph representations. Second, to encode both high-order and low-order pair-wise structural information from the candidate core subgraphs for prediction, DGIB4SL employs a motif-based GNN encoder [17]. Specifically, it uses 13 motif-based adjacency matrices to capture the high-order structure of a gene pair’s neighborhood, followed by a GNN with injective concatenation to combine motif-wise representations and produce the final representation of the core graph. We summarize our key contributions in the following.

- We employ the GIB principle to define a core subgraph, providing a principled alternative to attention weights, which often exhibit instability and bias toward frequent edges.
- We extend the GIB objective to handle data with multiple core subgraphs, resulting in DGIB, which serves as the objective for our DGIB4SL model.
- We use a motif-basd GNN encoder in DGIB4SL to capture both low- and high-order structures in node neighborhoods, ensuring reliable high-order structures in explanations.
- Experimental results demonstrate that our DGIB4SL outperforms state-of-the-art methods in both accuracy and explanation diversity.

## Related work

SL prediction methods can be categorized into three types: statistical inference methods, network-based methods, and supervised ML methods [18]. Statistical methods [19–22], such as ASTER [22], rely on predefined biological rules, which limit their applicability in complex systems due to strong underlying assumptions [23–25]. Network-based approaches [26–28], such as iMR-gMCSs [28], improved reproducibility by analyzing pathway level interactions. However, their performance is often limited by noise and incomplete data. With advancements in ML, supervised techniques such as SVM [29] and RF [30], and their combination [31] have been developed to facilitate feature selection using manually crafted biological features. However, their dependency on manual feature engineering poses the risk of overlooking critical interactions. SL$^{2}$MF [23] advances SL prediction by decomposing SL networks into matrices, offering a structured approach. However, its reliance on linear matrix decomposition struggles to capture the inherent complexity of SL networks. To overcome these limitations, deep learning methods [32–41] are developed. For example, DDGCN [32], the first GNN-based model, employs GCNs with dual-dropout to mitigate SL data sparsity. Similarly, MPASL [39] improves gene representations by capturing SL interaction preferences and layer-wise differences on heterogeneous graphs. Although many SL gene pairs have been identified, few of them have been applied to cancer treatment. Understanding the underlying biological mechanisms is crucial for developing SL-based cancer therapies. Unfortunately, most ML models lack the capability to fully explain SL mechanisms. To address this, methods incorporating prior knowledge into the above models through KG have been proposed [2–5, 38]. Most of these methods utilize attention mechanisms to identify important edges [2, 3], paths [4], or factors (subsets of relational features) [5] within KG to explain the mechanisms underlying SL. For example, KR4SL [4] encodes structural information, textual semantic information, and sequential semantics to generate gene representations and leverages attention to highlight important edges across hops to form paths as explanations. Similarly, SLGNN [5] focuses exclusively on KG data for factor-based gene representation learning, where relational features in the KG constitute factors, and attention weights are used to identify the most significant ones. However, attention weights are often unstable, frequently assigning higher weights to frequent edges [10], and typically provide only a single explanation per sample. Additionally, these methods struggle to capture high-order structures for prediction. To address these issues, DGIB4SL replaces attention mechanisms with graph information bottlenecks (IBs) to identify key edges and employs motif-based encoders along with DPP to encode high-order structures and generate multiple explanations. For further details on explainability in GNNs, please refer to our Supplementary Materials.

## Preliminaries

### Notations and problem formulation

An undirected SL graph is denoted by $G^{\text{SL}} = (\mathcal{V}^{\text{SL}}, \mathcal{Y}^{\text{SL}}, X^{\text{SL}})$, with the set of nodes (or genes) $\mathcal{V}^{\text{SL}}$, the set of edges or SL interactions $\mathcal{Y}^{\text{SL}}$, and the node feature matrix $X^{\text{SL}} \in \mathbb R^{|\mathcal{V}^{\text{SL}}|\times d_{0}}$. In addition to the SL interactions, we also have external knowledge about the functions of genes. We represent this information as a directed KG $G^{\text{KG}} = \{(h, r, t) | h, t \!\!\in \!\! \mathcal{V}^{\text{KG}}, r \!\!\in \!\! \mathcal{R}^{\text{KG}}\}$ and let $X^{\text{KG}}$ denote the node features associated with $G^{\text{KG}}$, where $\mathcal{V}^{\text{KG}}$ is a set of entities, and $\mathcal{R}^{\text{KG}}$ is a set of relations. To achieve the goals outlined later, we define $G = (A, X, E)$, where $A$ is the adjacency matrix, $X \in \mathbb R^{|\mathcal{V^{\text{SL}}} \cup \mathcal{V}^{\text{KG}}| \times d_{0}}$ is the node feature matrix, and $E \in \mathbb R^{|\mathcal{R}^{\text{KG}} \cup \{\hbox{``SL''}\}|\times d_{1}}$ is the edge feature matrix. Graph $G$ represents the directed *joint graph* of $G^{\text{SL}}$ and $G^{\text{KG}}$, constructed by mapping genes from $G^{\text{SL}}$ to entities in $G^{\text{KG}}$, and adding edges labeled “SL” for corresponding gene pairs based on $G^{\text{SL}}$. We use $(T)_{ij}$ to represent the element at the $i$th row and the $j$th column of a matrix $T$, and $(T)_{i*}$ to represent the $i$th row of the matrix. A comprehensive list of the mathematical notations used in this paper is provided in Table S1 in the Supplementary Materials.

In this paper, we investigate the problem of interpretable SLprediction, which aims to extract a local subgraph around two target genes for link prediction, potentially in an end-to-end fashion. Formally, given the joint graph $G$ that combines the SL graph and the KG, and a pair of genes $u$ and $v$, we first collect their $t$-hop neighborhoods from $G$ for each gene, $\mathcal{N}_{t}(u) = \{s|\text{dist}(s, u) \le t\}$, $\mathcal{N}_{t}(v) = \{s|\text{dist}(s, v) \le t\}$, where $\text{dist}(\cdot , \cdot )$ denotes the shortest distance between two nodes. We then take the intersection of nodes between their neighborhoods to construct a pairwise **enclosing graph** [3] $G^{uv} = \{(i, r, j)| i, j \in \mathcal{N}_{t}(u) \cap \mathcal{N}_{t}(v), r\in \mathcal{R}^{\text{KG}}\cup \{\hbox{``SL''}\}\}$. Our goal is to learn a function $f: \{G^{uv}|u,v \!\in \! \mathcal{V}^{\text{SL}}\} \!\to \! \{\widetilde{G}^{uv}|\widetilde{G}^{uv} \subseteq G^{uv}\}$, which maps the enclosing graphs $G^{uv}$ to optimized subgraph $\widetilde G^{uv}$, and learn a binary classifier $g_\theta (\widetilde G^{uv})$ parameterized by $\theta $ for SL prediction based on the optimized subgraph $\widetilde G^{uv}$.

### Information bottleneck

In ML, it is crucial to determine which parts of the input data should be preserved and which should be discarded. IB [42] offers a principled approach for addressing this challenge by compressing the source random variable to keep the information relevant for predicting the target random variable and discarding target-irrelevant information.

Definition 1 (IB). Given random variables $Q$ and $Y$, the IB principle aims to compress $Q$ to a bottleneck random variable $B$ while keeping the information relevant for predicting $Y$:

(1) \begin{align*}& \min_{B} \underbrace{-I(Y;B)}_{\text{Prediction}} + \underbrace{\beta I(Q;B)}_{\text{Compression}},\end{align*}

where $\beta> 0$ is a Lagrangian multiplier to balance the two mutual information terms.

Recently, the IB principle has been applied to learn a bottleneck graph named IB-Graph for the input graph [12], which keeps *minimal sufficient information* in terms of the graph’s data. In our context of SL prediction, the IB-Graph is defined as follows.

Definition 2 (IB-Graph). For an enclosing graph $G^{uv} = (A^{uv},$  $X^{uv}, E^{uv})$ around a pair of genes $u$ and $v$ and the associated label information $Y$, the optimal subgraph $\widetilde G^{uv} = (\widetilde A^{uv}, \widetilde X^{uv})$ found by IB principle is called IB-Graph if

(2) \begin{align*}& \widetilde G^{uv} = \underbrace{\arg\min_{\widetilde G^{uv}} -I(Y;\widetilde G^{uv}) + \beta I(G^{uv};\widetilde G^{uv})}_{\text{Graph Information Bottleneck, GIB}},\end{align*}

where $\widetilde A^{uv}$ and $\widetilde X^{uv}$ are the task-relevant adjacency matrix and the node feature matrix of $\widetilde G^{uv}$, respectively.

Intuitively, GIB (Equation 2) aims to learn the core subgraph of the input graph $G^{uv}$, discarding information from $G^{uv}$ by minimizing the mutual information $I(G^{uv}; \widetilde G^{uv})$, while preserving target-relevant information by maximizing the mutual information $I(Y;\widetilde G^{uv})$.

## Methods

### Overview

**Figure 2:**
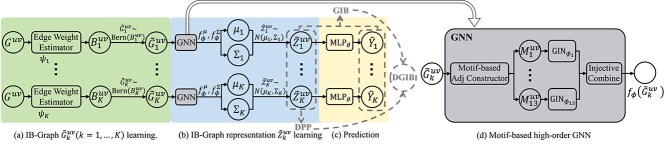
Overview of DGIB4SL. DGIB4SL takes the enclosing graph data $G^{uv} = (A^{uv}, X^{uv}, E^{uv})$ around genes $u$ and $v$ as inputs, throughout the phases (a),(b), and (c), and outputs the interaction confidence of the gene pair $(u, v)$ and $K$ IB-graphs $\widetilde G^{uv}_{1}$,..., $\widetilde G^{uv}_{K}$ that captures the high-order graph structure. In phase (a), an IB-graph $\widetilde G^{uv}_{k}$ is generated by injecting random noise to select important edges, with edge weights $ B^{uv}_{k} $ estimated from $ G^{uv} $ using the edge weight estimation module (Eq. S11). $B^{uv}_{k}$ serves as the parameter for a multidimensional Bernoulli distribution, from which an adjacency matrix of $\widetilde G^{uv}_{k}$ is sampled. In phase (b), IB-graph representations are learned via variational estimation. Each IB graph data $\widetilde G^{uv}_{k}$ is passed through the same motif-based GNN $ f_\phi $ (Eq. 10) to obtain a distribution from which a representation $ \widetilde Z^{uv}_{k} $ is sampled. The motif-based GNN, shown in subfigure (d), projects the IB-graph into 13 motif-based matrices. Each motif-based matrix $M^{uv}_{k}$ is processed by a different GIN encoder to produce motif-wise representations, which are then concatenated (Eq. 15). In phase (c), each IB-graph representation is passed through an Multilayer Perceptrons (MLP)-based classifier to make $ K $ predictions (Eq. 11). During training, the representations and predictions are used to compute DPP and GIB, which are jointly optimized in DGIB4SL.

In this section, we present DGIB4SL, an interpretable SL prediction framework that incorporates a diversity constraint using DPP and GIB objective to generate multiple explanations called IB-graphs for the same gene pair. The framework consists of three key components: IB objective formulation, motif-based DGIB estimation, and prediction. First, we introduce our DGIB objective and derive its tractable upper bound. Next, given that most of the existing IB estimation approaches fail to capture high-order structure information, we propose a novel motif-based DGIB estimation method, which involves three phases: IB-graph learning through random noise injection to select significant edges, GRL, and prediction, as shown in Fig. 2(a)–(c). In the GRL phase, we employ the motif-wise representation learning method [17] to implement the GNN module in Fig. 2(b), enabling the capture of high-order structures in IB-graph, as illustrated in Fig. 2(d).

### Diverse graph information bottleneck

We now present our main result, which demonstrates how to generate $K \ge 1$ different IB-graphs for any gene pair $(u, v)$, denoted as $\{\widetilde G^{uv}_{1},..., \widetilde G^{uv}_{K}\}$. We first reduce this problem to a special case of the *subset selection problem* where diversity is preferred, i.e. the problem of balancing two aspects: (i) each selected subgraph should satisfy the definition of an IB-Graph; and (ii) the selected subgraphs should be diverse as a group so that the subset is as informative as possible.

DPP [16] is an elegant and effective probabilistic model designed to address one key aspect of the above problem: diversity. Formally, let ${\mathcal{G}}^{uv}$ denote the set of all possible subgraphs of a graph $G^{uv}$. A point process $P$ defined on the ground set ${\mathcal{G}}^{uv}$ is a probability measure over the power set of ${\mathcal{G}}^{uv}$. $P$ is called a DPP if, for any subset $\{\widetilde G^{uv}_{1},..., \widetilde G^{uv}_{K}\} \subseteq \mathcal{G}^{uv}$, the probability of selecting this subset is given by


(3) \begin{align*}& P(\{\widetilde G^{uv}_{1},..., \widetilde G^{uv}_{K}\}) \propto \text{det}(L^{uv}),\end{align*}

where $\text{det}(\cdot )$ represents the determinant of a given matrix and $L^{uv} \in \mathbb R^{K \times K}$ is a real, positive semidefinite matrix and thus there exists a matrix $U\in \mathbb R^{K \times d_{3}}$ such that


(4) \begin{align*}& L^{uv} = UU^{\mathrm{T}}, (U)_{k} = \text{GRL}(\widetilde G^{uv}_{k}),\end{align*}

where $(U)_{k} \in \mathbb R^{d_{3}}$ is the graph representation of the $k$th IB-graph $\widetilde G^{uv}_{k}$ and $\text{GRL}$ denotes the GRL module. More details about the $\text{GRL}$ module and intuitions about the ability of Equation 3 to measure diversity are provided in Eqs. 9–12 and Supplementary Materials, respectively.

To learn $K$ different subgraphs from the enclosing graph $G^{uv}$ for the gene pair $(u,v)$ that balance diversity with the IB-graph definition, we introduce the Diverse Graph Information Bottleneck (DGIB) objective function, formulated as follows:


(5) \begin{align*}& \min_{\widetilde G^{uv}_{1},..., \widetilde G^{uv}_{K}} \frac{1}{K}\sum^{K}_{k=1} \underbrace{-I(Y;\widetilde G^{uv}_{k}) + \beta_{1} I(G^{uv};\widetilde G^{uv}_{k})}_{\text{Graph Information Bottleneck}} - \beta_{2} \underbrace{\text{det}(L^{uv})}_{\text{DPP}},\end{align*}

where $\beta _{2}> 0$ is a Lagrangian multiplier to trade off GIB and DPP terms. Intuitively, the GIB term focuses on learning multiple IB-graphs from the input graph $G^{uv}$, while the DPP term ensures that these IB-graphs are as different as possible.

Due to the non-Euclidean nature of graph data and the intractability of mutual information, it is challenging to optimize the DGIB objective in Equation 5 directly. Therefore, we adopt the approach of Sun *et al*. [43] to derive tractable variational upper bounds for $-I(Y;\widetilde G^{uv}_{k})$ and $I(G^{uv};\widetilde G^{uv}_{k})$:


(6) \begin{align*} \min_{\widetilde G^{uv}_{1},..., \widetilde G^{uv}_{K}} &\frac{1}{K}\sum^{K}_{k=1} \underbrace{{-\mathbb E_{Y, \widetilde G^{uv}_{k}} \Big[\log q_\theta(Y|\widetilde G^{uv}_{k})\Big]}}_{\text{Upper bound of } {-I(Y;\widetilde G^{uv}_{k})}}\nonumber \\ &+ \beta_{1}\frac{1}{K}\sum^{K}_{k=1} \underbrace{\mathbb E_{G^{uv}} [D_{\text{KL}}(q_\phi(\widetilde G^{uv}_{k}|G^{uv})||q(\widetilde G^{uv}_{k})]}_{\text{Upper bound of } {I(G^{uv};\widetilde G^{uv}_{k})}}\nonumber \\ &- \beta_{2} \text{det}(L^{uv}). \end{align*}

Detailed proof of Equation 6 is given in Supplementary Materials.

Remark 1. Each explanation or IB-graph generated by DGIB4SL consists of a single core subgraph rather than a combination of multiple core subgraphs. In datasets with tens of thousands of gene pairs, as used in our experiments, individual core subgraphs are more likely to be shared across different enclosing graphs than combinations of multiple core subgraphs. This is because the probability of a specific combination being repeatedly shared decreases exponentially with its complexity. In contrast, the structural simplicity of individual core subgraphs makes them more likely to be shared. Minimizing the compression term in DGIB allows DGIB4SL to select individual core subgraphs with higher shared frequency.

### High-order motif-based DGIB estimation

We now address another key question of this work: how to compute the DGIB upper bound in Equation 6 without losing the high-order information, which is crucial for generating trustworthy explanations. For instance, in a KG, a gene’s functional relevance often depends on high-order structures, such as cooperative pathways or shared regulatory targets among its neighbors. Ignoring these structures can result in misleading explanations. To overcome this, we propose a novel high-order motif-based DGIB estimation method DGIB4SL.

#### Mutual information estimation

We first outline the general procedure for estimating the DGIB upper bound defined in Equation 6, which is largely analogous to previous work [43, 44]. This procedure involves learning the $k$th IB-graph $\widetilde G^{uv}_{k}$ from the enclosing graph $G^{uv}$ and driving its representation $\widetilde Z^{uv}_{k} \in \mathbb R^{d_{3}}$ through a GRL function ($\text{GRL}$), such that $\widetilde Z^{uv}_{k} = \text{GRL}(\widetilde G^{uv}_{k})$, assuming no information is lost during this transformation. Under this assumption, $I(Y;\widetilde Z_{k}^{uv}) \approx I(Y;\widetilde G_{k}^{uv})$, $I(G^{uv};\widetilde Z_{k}^{uv}) \approx I(G^{uv};\widetilde G_{i}^{uv})$. Consequently, the DGIB upper bound, which DGIB4SL aims to minimize, is expressed as


(7) \begin{align*} \min_{\widetilde G^{uv}_{1},..., \widetilde G^{uv}_{K}} -&\frac{1}{K}\sum^{K}_{k=1} \mathbb E_{Y, \widetilde G^{uv}_{k}} \Big[\log q_\theta(Y| \widetilde Z^{uv}_{k})\Big] - \beta_{2} \text{det}(L^{uv}) \nonumber \\ &+ \beta_{1}\frac{1}{K}\sum^{K}_{k=1} \mathbb E_{G^{uv}} [D_{\text{KL}}(q_\phi( \widetilde Z^{uv}_{k}|G^{uv})||q(\widetilde Z^{uv}_{k})] \end{align*}

To calculate Equation 7, we follow a two-step process. In **Step 1**, we estimate a IB-graph $\widetilde G_{k}^{uv}$ based on all the subgraphs from $G^{uv}$. In **Step 2**, we implement the $\text{GRL}$ function to infer the graph representation $\widetilde Z_{k}^{uv}$ of $\widetilde G_{k}^{uv}$ and feed $\widetilde Z_{k}^{uv}$ into Equation 7.

(**Step 1: IB-graph $\widetilde G_{k}^{uv}$ learning**) We compress the information of $G^{uv} = (A^{uv}, X^{uv}, E^{uv})$ via noise injection to estimate the $k$th IB-graph $\widetilde G^{uv}_{k} = (\widetilde A^{uv}_{k}, \widetilde X^{uv}_{k})$. To construct $\widetilde A^{uv}_{k}$, we model all potential edges of the subgraph as mutually independent Bernoulli random variables. The parameters of these variables are determined by the learned important weights $B_{k}^{uv} \in \mathbb R^{|\mathcal{V}^{uv}|\times |\mathcal{V}^{uv}|}$, where $\mathcal{V}^{uv}$ denotes the set of entities in $G^{uv}$:


(8) \begin{align*}& \widetilde A^{uv}_{k} = \bigcup_{i,j \in \mathcal{V}^{uv}}\left\{(\widetilde A^{uv}_{k})_{i, j} \sim \operatorname{Bernoulli}\left((B_{k}^{uv})_{i,j}\right)\right\},\end{align*}

where $(B_{k}^{uv})_{i,j}$ represents the importance weight or sampling probability for the entity pair $(i,j)$. The computation of $B_{k}^{uv}$ (corresponding to $\psi _{k}$ in Fig. 1) is jointly optimized with relational graph learning, following the approach of Wang *et al*. [45]. Further details are provided in Supplementary Materials. To sample the IB-graph, we employ the concrete relaxation [46] for the Bernoulli distribution. Additionally, we construct $\widetilde X_{k}^{uv}$ to be the same as $X^{uv}$ since no nodes are removed during the construction of $\widetilde A_{k}^{uv}$. An example of the IB-graph construction is provided in the Supplementary Materials.

(**Step 2: IB-GRL and prediction**) Using the previously constructed $\widetilde G_{k}^{uv} = (\widetilde A_{k}^{uv}, \widetilde X_{k}^{uv})$, we compute the prediction, diversity, and KL terms in Equation 7 by implementing $\widetilde Z_{k}^{uv} = \text{GRL}(\widetilde G_{k}^{uv})$ through variational inference. For the KL term $D_{\text{KL}}(q_\phi ( \widetilde Z^{uv}_{k}|G^{uv})||q(\widetilde Z^{uv}_{k}))$, we treat the prior $p(\widetilde Z^{uv}_{k})$ and the posterior $q_\phi (\widetilde Z^{uv}_{k}|G^{uv})$ as parametric Gaussians and thus this term has an analytic solution:


(9) \begin{align*}& \begin{aligned} &p(\widetilde Z_{k}^{uv}):= N(\mu_{0}, \Sigma_{0}),\\ &q_\phi(\widetilde Z_{k}^{uv}|G^{uv}):= N(f^\mu_{\phi}(\widetilde A_{k}^{uv}, \widetilde X_{k}^{uv}), f^\Sigma_\phi(\widetilde A_{k}^{uv}, \widetilde X_{k}^{uv})), \end{aligned}\end{align*}

where the outputs $f^\mu _\phi (\cdot ) \in \mathbb R^{d_{3}}$ and $f^\Sigma _\phi (\cdot ) \in \mathbb R^{d_{3} \times d_{3}}$ represent the mean vector and the diagonal covariance matrix of the distribution for the graph embedding $\widetilde Z_{k}^{uv} \in \mathbb R^{d_{3}}$ of $\widetilde G_{k}^{uv}$, respectively. We model $f_\phi (\cdot )$ as a GNN parameterized by the weights $\phi $ with $2d_{3}$-dimensional output and a readout or pooing operator. The first $d_{3}$ dimensions of this GNN’s output correspond to $f^\mu _\phi (\cdot )$, while the remaining $d_{3}$ dimensions correspond to $f^\Sigma _{\phi }(\cdot )$, formally expressed as


(10) \begin{align*}& \left(f^\mu_{\phi}(\widetilde A_{k}^{uv}, \widetilde X_{k}^{uv}), f^\Sigma_{\phi}(\widetilde A_{k}^{uv}, \widetilde X_{k}^{uv})\right) = \mathrm{readout}\left(\mathrm{GNN}\left(\widetilde A_{k}^{uv}, \widetilde X_{k}^{uv};\phi\right)\right).\end{align*}

We treat $p(\widetilde Z_{k}^{uv}):= N(\mathbf{0}, I)$ as a fixed $d_{3}$-dimensional spherical Gaussian distribution. To compute the prediction term $\mathbb E_{Y, \widetilde G^{uv}_{k}} [\log q_\theta (Y| \widetilde Z^{uv}_{k})]$, we adopt the equivalent cross entropy loss function $L^{\mathrm{CE}}$. The conditional distribution $q_\theta (Y| \widetilde Z^{uv}_{k})$ is implemented using a two-layer perceptron in this work, parameterized by trainable weights $\theta $, as described below.


(11) \begin{align*}& -\mathbb E_{Y, \widetilde G_{k}^{uv}} \Big[\log q_\theta(Y| \widetilde Z_{k}^{uv})\Big] = L^{\mathrm{CE}}(Y, \mathrm{MLP}(\widetilde Z_{k}^{uv};\theta)).\end{align*}

Finally, for the diversity term $\text{det}(L^{uv})= \text{det}(UU^{\mathrm{T}})$ (Equation 7, Equation 4), the matrix $U$ is constructed by arranging the $K$ IB-graph representations as its rows. Specifically,


(12) \begin{align*}& U = (\widetilde Z_{1}^{uv},..., \widetilde Z_{K}^{uv})^{\mathrm{T}}.\end{align*}

#### Generating high-order graph representation via motif

Most methods struggle to satisfy the no-information-loss assumption of the above mutual information estimation framework, since their GNN implementation in Eq.10 often fails to capture the high-order structure of the estimated explanation. Inspired by MGNN [17], we reduce this to a problem of enhancing the model’s representation power beyond the one-dimensional Weisfeiler–Leman ($1$-WL) graph isomorphism test [47]. Specifically, the 1-WL test distinguishes graph structures by iteratively compressing node neighborhood information into unique labels, making it a widely recognized tool for evaluating the expressive power of GNNs [17, 47, 48].

We first formalize three key definitions underlying our approach, starting with the notion of a network motif.

Definition 3 (Network motif). A motif is a connected graph of $d_{5}$ nodes ($d_{5}> 1$), with a $d_{5} \times d_{5}$ adjacency matrix $C$ containing binary elements $\{0, 1\}$.

**Figure 3:**
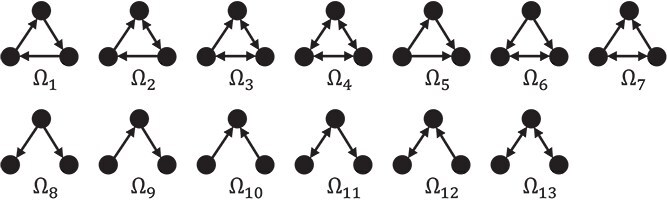
All three-node motifs in a directed and unweighted graph.

Let $\Omega _{i}$ denote different motifs and $C_{i}$ represent the corresponding associated matrices. An example of all possible three-node motifs is shown in Fig. 3. Chen *et al*. [17] demonstrated that three-node motifs are sufficiently expressive to capture graph structures. Thus, we only use motifs with three nodes in this work.

Definition 4 (Motif set). The motif set of a three-node motif $\Omega _{i}$ in a directed graph $G=(A,X)$ is defined by

(13) \begin{align*}& \mathcal{M}_{i} = \{ V | V \in \mathcal{V}^{3}, A^{V} = C_{i}\},\end{align*}

where $V$ is a tuple containing three node indices and $A^{V}$ is the $d_{5} \times d_{5}$ adjacency matrix of the subgraph induced by $V$.

For example, the motif set of $\Omega _{5}$ in Fig. 1 can be $\{(\text{HR}, \text{Trapped} \text{replication fork}, \text{DNA damage}),$  $(\text{HR}, \text{DSB}, \text{DNA}$  $\text{damage})\}$. Based on the motif set, we define the operator $\text{set}(\cdot )$ to transform an ordered tuple into an unordered set, e.g. $\mathrm{set}((v_{1},..., v_{3})) = \{v_{1},..., v_{3}\}$. Using this operator, the motif-based adjacency matrix is defined as follows.

Definition 5 (Motif-based adjacency matrix). For a graph $G=(A,X)$, a motif-based adjacency matrix $M_{i}$ of $G$ in terms of a given motif $\Omega _{i}$ is defined by

(14) \begin{align*}& (M_{i})_{j,l} = \sum_{V \in \mathcal{M}_{i}} \mathbb I( \{j,l\}\subset \mathrm{set}(V)).\end{align*}

Intuitively, $(M_{i})_{j,l}$ denotes the number of times nodes $j$ and $l$ are connected through an element of the given motif set $\mathcal{M}_{i}$. The roles of these definitions will be discussed in Equation 15.

**Table 1:** Performance of various methods in terms of NDCG and Recall under five-fold cross-validation. Values in parentheses indicate paired $t$-test $P$-values comparing baselines with DGIB4SL.

	NDCG@10	NDCG@50	Recall@10	Recall@50
GRSMF	0.2844 ($2.06 \times 10^{-7}$)	0.3153 ($9.20 \times 10^{-9}$)	0.3659 ($8.04 \times 10^{-7}$)	0.4460 ($3.07 \times 10^{-4}$)
SL$^{2}$MF	0.2807 ($7.79 \times 10^{-8}$)	0.3110 ($7.49 \times 10^{-8}$)	0.2642 ($8.44 \times 10^{-7}$)	0.3401 ($2.95 \times 10^{-7}$)
CMFW	0.2390 ($1.24 \times 10^{-7}$)	0.2744 ($7.16 \times 10^{-7}$)	0.3257 ($2.15 \times 10^{-6}$)	0.4097 ($4.14 \times 10^{-5}$)
DDGCN	0.1568 ($1.94 \times 10^{-7}$)	0.1996 ($6.74 \times 10^{-8}$)	0.2379 ($7.61 \times 10^{-9}$)	0.3447 ($2.76 \times 10^{-5}$)
GCATSL	0.2642 ($3.99 \times 10^{-7}$)	0.2976 ($1.24 \times 10^{-6}$)	0.3363 ($5.91 \times 10^{-6}$)	0.4203 ($3.67 \times 10^{-4}$)
SLMGAE	0.2699 ($6.63 \times 10^{-7}$)	0.3160 ($1.19 \times 10^{-6}$)	0.3198 ($1.26 \times 10^{-5}$)	0.4421 ($1.26 \times 10^{-3}$)
MGE4SL	0.0028 ($2.66 \times 10^{-9}$)	0.0071 ($4.89 \times 10^{-9}$)	0.0020 ($1.35 \times 10^{-8}$)	0.0085 ($6.13 \times 10^{-9}$)
PTGNN	0.2358 ($1.07 \times 10^{-7}$)	0.2740 ($6.66 \times 10^{-7}$)	0.3361 ($9.43 \times 10^{-6}$)	0.4323 ($1.96 \times 10^{-4}$)
KG4SL	0.2505 ($1.04 \times 10^{-6}$)	0.2853 ($1.05 \times 10^{-7}$)	0.3347 ($4.40 \times 10^{-7}$)	0.4253 ($2.48 \times 10^{-5}$)
PiLSL	0.5166 ($3.32 \times 10^{-5}$)	0.5175 ($3.28 \times 10^{-5}$)	0.3970 ($3.99 \times 10^{-6}$)	0.4021 ($3.82 \times 10^{-6}$)
NSF4SL	0.2279 ($2.65 \times 10^{-6}$)	0.2706 ($4.50 \times 10^{-7}$)	0.3526 ($2.96 \times 10^{-5}$)	0.4624 ($1.14 \times 10^{-3}$)
KR4SL	0.5105 ($2.19 \times 10^{-5}$)	0.5248 ($5.79 \times 10^{-5}$)	0.4131 ($6.97 \times 10^{-6}$)	0.4135 ($5.62 \times 10^{-6}$)
SLGNN	0.1468 ($4.49 \times 10^{-8}$)	0.2004 ($1.85 \times 10^{-7}$)	0.2154 ($1.90 \times 10^{-7}$)	0.3717 ($1.26 \times 10^{-4}$)
DGIB4SL	**0.5760**	**0.5766**	**0.5233**	**0.5280**

To generate graph embeddings with greater expressive power than the $1$-WL test, Chen *et al*. [17] demonstrated that associating node or graph embeddings with different motif structures and combining these embeddings using an injective function effectively captures high-order and low-order graph structure. Specifically, we use a two-layer GIN [47] as the underlying GNN and employ different GINs to encode the structure of 13 motifs in $\widetilde G^{uv}$, producing node embeddings through motif-based adjacency matrices. Then, the motif-wise embeddings are combined via injective concatenation. Mathematically, we construct the GNN module in Eq.10 as


(15) \begin{align*}& \mathrm{GNN}(\widetilde A_{k}^{uv}, \widetilde X_{k}^{uv};\phi):= \|_{i=1}^{13} \mathrm{GIN}( M^{uv}_{i}, \widetilde X^{uv}_{k};\phi_{i}),\end{align*}

where $M^{uv}_{i}$ are the motif-based adjacency matrix of $\widetilde G^{uv} = (\widetilde A_{k}^{uv}, \widetilde X_{k}^{uv})$ in terms of a given motif $\Omega _{i}$ and ∥ denotes a concatenation function. In summary, Equation 15 preserves high-order and low-order pair-wise structural information when calculating DGIB, enhancing the reliability of the high-order structure in an IB-graph.

## Results

### Experimental setup

#### Datasets and baselines

To evaluate the effectiveness of our DGIB4SL, we utilized the dataset provided by the Synthetic Lethality Benchmark (SLB) [49]. The dataset is collected from SynLethDB 2.0, a comprehensive repository of SL data, and includes 11 types of entities and 27 relationships. It contains 35 913 human SL gene pairs involving 9845 genes, along with a KG named SynLethKG, which comprises 54 012 nodes and 2233 172 edges. Additional details on SynLethKG can be found in Tables S2–S3 in Supplementary Materials.

We evaluated two categories of methods, selecting 13 recently published methods. These include three matrix factorization (MF)-based methods: GRSMF [25], SL2MF [23], and CMFW [24], and 10 GNN-based methods: DDGCN [32], GCATSL [35], SLMGAE [36], MGE4SL [34], PTGNN [37], KG4SL [2], PiLSL [3], NSF4SL [38], KR4SL [4], and SLGNN [5]. Among these, KG4SL, PiLSL, NSF4SL, KR4SL, and SLGNN integrate KGs into the generation of node representations. Detailed descriptions of these baselines can be found in Supplementary Materials.

#### Implementation details

We evaluated our method using five-fold cross-validation by splitting the gene pairs and using four ranking metrics: Normalized Discounted Cumulative Gain (NDCG@C), Recall@C, Precision-@C, and Mean Average Precision (MAP@C). NDCG@C measures the positioning of known SL gene pairs within the model’s predicted list, while Recall@C and Precision@C assess the model’s ability to identify relevant content and rank the top C results accurately, respectively. MAP@C provides a comprehensive evaluation by combining precision and ranking across multiple queries, averaging the precision at each relevant prediction up to the Cth position. In this study, we evaluated these metrics using the top C=10 and top C=50 predictions. The coefficients $\beta _{1}$ and $\beta _{2}$ in Equation 6 were set $\beta _{1} = \beta _{2} = 10^{-4}$. More details on data preprocessing, hyperparameters settings for DGIB4SL, and baseline implementations are provided in Supplementary Materials.

### Performance evaluation

We evaluated the empirical performance of DGIB4SL against state-of-the-art baselines, as summarized in Table 1 and Table 2. Baseline performance was referenced from the public leaderboards provided by SLB [49], except for KR4SL, which was based on our experimental results. As shown in Table 1 and Table 2, DGIB4SL consistently outperformed all baselines on the SynLethDB 2.0 dataset [50]. Specifically, KR4SL achieved the second-best performance on NDCG@50, Recall@10, Precision@10, Precision@50, MAP@10, and MAP@50, while PiLSL and NSF4SL achieved the second-best performance on NDCG@10 and Recall@50, respectively. Our DGIB4SL further improved over KR4SL by 9.9$\%$, 26.7$\%$, 10.6$\%$, 8.0$\%$, 6.0$\%$, and 5.5$\%$ on NDCG@50, Recall@10, Precision@10, Precision@50, MAP@10, and MAP@50, respectively, and outperformed PiLSL and NSF4SL by 11.5$\%$ and 14.2$\%$ in NDCG@10 and Recall@50, respectively. From these results, we draw the following conclusions: (i) the competitive performance of DGIB4SL and KG-based baselines highlights the value of KGs in providing biological context for gene related label prediction. (ii) The integration of motifs significantly enhances model performance by expanding the receptive field and encoding high-order edges into predictions effectively.

**Table 2:** Performance of various methods in terms of Precision and MAP under five-fold cross-validation.

	Precision@10	Precision@50	MAP@10	MAP@50
GRSMF	0.3683 ($1.02 \times 10^{-5}$)	0.4461 ($1.42 \times 10^{-4}$)	0.2568 ($7.15 \times 10^{-7}$)	0.2521 ($9.92 \times 10^{-8}$)
SL$^{2}$MF	0.2694 ($3.53 \times 10^{-7}$)	0.3407 ($3.45 \times 10^{-6}$)	0.2861 ($1.35 \times 10^{-7}$)	0.2769 ($1.68 \times 10^{-7}$)
CMFW	0.3267 ($8.57 \times 10^{-7}$)	0.4098 ($4.37 \times 10^{-5}$)	0.2043 ($5.65 \times 10^{-7}$)	0.2069 ($1.13 \times 10^{-6}$)
DDGCN	0.2385 ($1.60 \times 10^{-6}$)	0.3447 ($1.41 \times 10^{-5}$)	0.1280 ($6.26 \times 10^{-9}$)	0.1321 ($3.74 \times 10^{-8}$)
GCATSL	0.3372 ($5.36 \times 10^{-6}$)	0.4204 ($1.03 \times 10^{-3}$)	0.2354 ($8.96 \times 10^{-7}$)	0.2382 ($7.66 \times 10^{-8}$)
SLMGAE	0.3222 ($3.58 \times 10^{-6}$)	0.4422 ($7.76 \times 10^{-4}$)	0.2514 ($2.10 \times 10^{-7}$)	0.2469 ($9.86 \times 10^{-7}$)
MGE4SL	0.0022 ($1.31 \times 10^{-8}$)	0.0085 ($8.87 \times 10^{-9}$)	0.0018 ($3.17 \times 10^{-9}$)	0.0024 ($1.58 \times 10^{-8}$)
PTGNN	0.3372 ($2.26 \times 10^{-6}$)	0.4324 ($1.48 \times 10^{-4}$)	0.1948 ($3.46 \times 10^{-7}$)	0.1975 ($1.80 \times 10^{-7}$)
KG4SL	0.3357 ($4.68 \times 10^{-6}$)	0.4254 ($1.43 \times 10^{-4}$)	0.2175 ($1.50 \times 10^{-6}$)	0.2208 ($1.04 \times 10^{-6}$)
PiLSL	0.4098 ($3.71 \times 10^{-6}$)	0.4035 ($3.84 \times 10^{-6}$)	0.5153 ($4.95 \times 10^{-4}$)	0.5149 ($2.40 \times 10^{-3}$)
NSF4SL	0.3563 ($1.34 \times 10^{-5}$)	0.4626 ($2.69 \times 10^{-4}$)	0.1881 ($1.85 \times 10^{-6}$)	0.1818 ($3.65 \times 10^{-6}$)
KR4SL	0.4845 ($1.50 \times 10^{-4}$)	0.4901 ($4.75 \times 10^{-4}$)	0.5175 ($6.82 \times 10^{-4}$)	0.5200 ($5.33 \times 10^{-3}$)
SLGNN	0.2172 ($1.86 \times 10^{-6}$)	0.3718 ($5.13 \times 10^{-5}$)	0.1259 ($1.16 \times 10^{-7}$)	0.1252 ($2.34 \times 10^{-8}$)
DGIB4SL	**0.5359**	**0.5294**	**0.5485**	**0.5484**

### Explanation evaluation

#### Qualitative analysis

**Figure 4:**
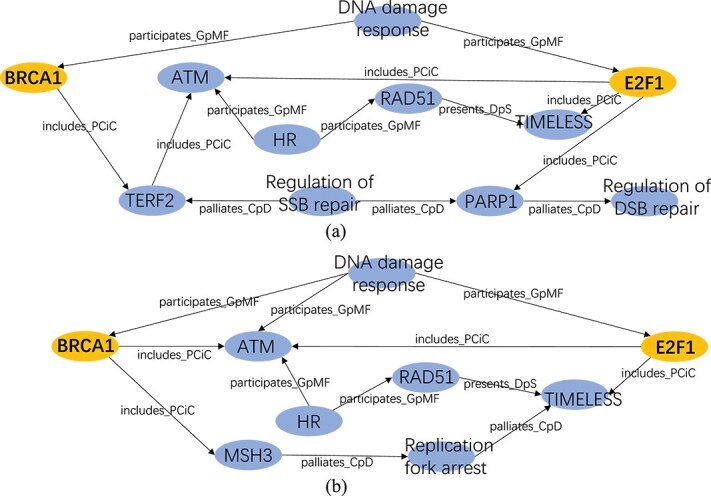
Two explanations learned from our DGIB4SL provide different insights into the biological mechanisms underlying SL of the same gene pair (BRCA1, E2F1). For details on the edge nomenclature, please refer to Table S4 in Supplementary Materials.

Leveraging the DGIB mechanism (Equation 6), our DGIB4SL not only predicts SL interactions but also provides $K \ge 1$ explanations that reveal the biological mechanisms underlying the predictions for the same gene pair. For this case study, we selected the SL pair BRCA1 and E2F1 from the test data, where the predicted interaction between BRCA1 and E2F1 matched the actual label. To remove unimportant edges from the enclosing core graphs of (BRCA1, E2F1), we applied edge sampling probabilities with thresholds 0.58 and 0.76 for the first and second core subgraphs distribution (Equation 8), respectively. Edges with probabilities exceeding these thresholds ($(B^{uv}_{1})_{i,j}> 0.58$, $(B^{uv}_{2})_{i,j}> 0.76$) were retained. The filtered core graphs are shown in Fig. 4(a) and (b).

We first analyzed the first core subgraph (Fig. 4(a)). The first core subgraph highlights two key mechanisms of SL between BRCA1 and E2F1: (1) homologous recombination (HR) deficiency due to BRCA1 mutation: pathway “BRCA1 $\xrightarrow{\text{includes}{\_}\text{PCiC}}$TERF2$\xrightarrow{\text{includes}{\_}\text{PCiC}}$ ATM $\xleftarrow{\text{participates}{\_}\text{GpMF}}$ HR$\xrightarrow{\text{participates}{\_}\text{GpMF}}$RAD51$\xrightarrow{\text{presents}{\_}\text{DpS}}$ TIMELESS” indicates that BRCA1 mutation inactivates the HR pathway. This leaves DSBs, converted from unresolved SSBs, unrepaired. (2) SSB repair pathway blockage: the pathways “E2F1$\xrightarrow{\text{includes}{\_}\text{PCiC}}$ PARP1$\xrightarrow{\text{palliates}{\_}\text{CpD}}$ Regulation of DSB repair” and “Regulation of SSB repair$\xrightarrow{\text{palliates}{\_}\text{CpD}}$ PARP1” demonstrate that E2F1 mutation weakens both SSB and DSB repair functions. These combined defects in SSB repair and HR result in unrepairable DNA damage, genomic instability, and ultimately cell death. Previous studies [51] have shown that E2F1 depletion impairs HR, disrupting DNA replication and causing DNA damage, further supporting these findings.

We then analyzed the second core subgraph (Fig. 4(b)). The second core subgraph identifies a different mechanism, centered around replication fork blockage, while maintaining the shared premise of HR repair pathway loss. Specifically, the pathway “E2F1$\xrightarrow{\text{includes}{\_}\text{PCiC}}$TIMELESS $\xleftarrow{\text{palliates}{\_}\text{CpD}}$Replication Fork Arrest” reveals that E2F1 mutation destabilizes replication forks, leading to stalled replication. TIMELESS, a downstream target of E2F1, plays a critical role in stabilizing replication forks during DNA replication stress.

#### Quantitative analysis

We evaluated the Infidelity [52] and Sparseness [53] (see supplementary materials for these metrics descriptions) and used the DPP to evaluate the diversity of explanations generated by DGIB4SL and other explainable SL prediction methods, including KG4SL, PiLSL, SLGNN, and KR4SL. To compare diversity, we introduced KR4SL$^{*}$, a variant of KR4SL with a multihead attention mechanism, since the explainable baselines (except for SLGNN) generate a single explanation using similar attention mechanisms. As shown in Table 3, DGIB4SL outperforms other methods in terms of Infidelity, Sparseness, and DPP. We draw the following conclusions:

- **Diversity**: despite using multihead attention, KR4SL$^{*}$ showed lower DPP values, indicating that multihead attention alone has a limited capacity for generating diverse explanations. SLGNN’s DPP performance is competitive, due to the inclusion of a distance correlation regularizer that encourages independent factor embeddings, indirectly enhancing diversity.
- **Sparsity**: baselines employing similar attention mechanisms showed comparable Sparseness values, except for SLGNN, which directly uses learnable weights to estimate the importance of different relational features.
- **Fidelity**: the Infidelity of attention-based methods is relatively low, possibly due to the inherent instability and high-frequency biases of attention mechanisms [6, 7].

**Table 3:** Comparison of attention weights in KG-Based SL prediction methods with explanations in DGIB4SL in terms of Fidelity, Sparsity, and Diversity. Symbols $\uparrow $ and $\downarrow $, respectively, represent that larger and smaller metric values are better.

	Infidelity$\downarrow $	Sparseness$\uparrow $	DPP$\uparrow $
KG4SL	$4.0\!\times \!10^{5}\ (2\!\times \!10^{-4})$	0.330 ($8\!\times \!10^{-4}$)	–
PiLSL	$5.7\!\times \!10^{5}\ (9\!\times \!10^{-3})$	0.340 ($4\!\times \!10^{-4}$)	–
SLGNN	$1.8\!\times \!10^{6}\ (1\!\times \!10^{-9})$	0.120 ($6\!\times \!10^{-5}$)	1.59 ($8\!\times \!10^{-4}$)
KR4SL	$\underline{5.8\!\times \!10^{4}}\ (2\!\times \!10^{-3})$	0.352 ($7\!\times \!10^{-4}$)	–
KR4SL$^{*}$	$\underline{5.8\!\times \!10^{4}}\ (1\!\times \!10^{-3})$	0.326 ($5\!\times \!10^{-4}$)	0.48 ($7\!\times \!10^{-4}$)
DGIB4SL	$\mathbf{6.2}\!\times \!\mathbf{10}^{\mathbf{3}}$	**0.463**	**1.67**

### Model analysis

#### Ablation study

**Figure 5:**
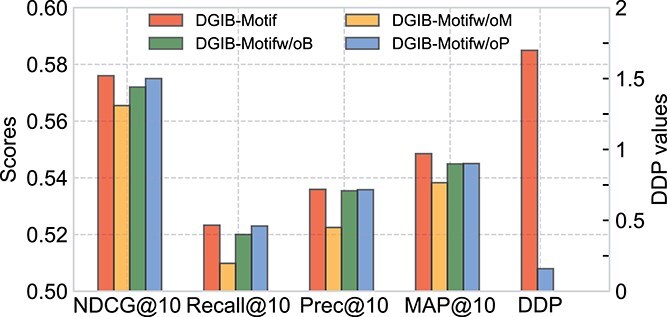
Ablation study of DGIB4SL for Motif, DGIB, and DPP on NDCG@10, Recall@10, Precision@10, MAP@10 (left Y-axis) and one diversity metric DPP (right Y-axis).

As illustrated in Fig. 2, the DGIB (Equation 6), DPP constraint (third line in Equation 6), and motif-based graph encoder (Equation 15) are key components of DGIB4SL. Based on these, we derived the following variants for the ablation study: (1) DGIB4SLw/oM: DGIB4SL without motif information, to assess the impact of motifs; (2) DGIB4SLw/oB: DGIB4SL without the DGIB objective (replacing it with an attention mechanism); and (3) DGIB4SLw/oP: DGIB4SL without the DPP constraint (essentially reducing the objective to GIB). To evaluate the contributions of motifs, DGIB and DPP, we compared DGIB4SL against these variants. As shown in Fig. 5, DGIB4SL consistently outperformed DGIB4SLw/oM across all metrics, highlighting the importance of incorporating high-order structures through motifs in SL prediction. Secondly, the performance of DGIB4SLw/oB was comparable with DGIB4SL on all metrics. This result is expected, since DGIB4SLw/oB can still extract label-related input information via attention mechanisms, even if this information may not always faithfully reflect the model’s behavior. Thirdly, DGIB4SLw/oP also achieved comparable performance to DGIB4SL. This is intuitive since, without DPP constraints, DGIB4SLw/oP may find $K$ similar explanations, which could overlap with one of the $K$ different explanations found by DGIB4SL. To further compare their explanations, we evaluated their diversity using the DPP measure, calculated as the determinant of $\widetilde G^{uv}_{1}$,..., $\widetilde G^{uv}_{K}$ (Eqs. 3 and 4). As shown in the two rightmost columns of Fig. 5, DGIB4SL produced significantly more diverse explanations compared with DGIB4SLw/oP.

#### Convergence analysis

**Figure 6:**
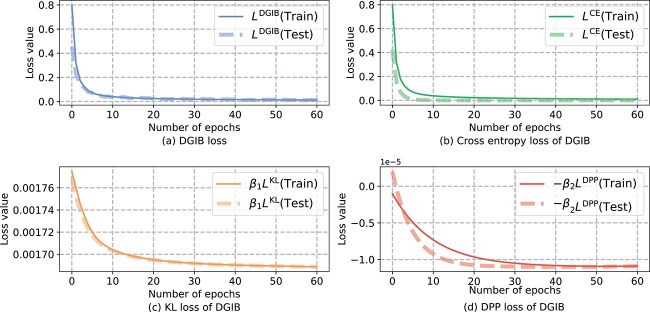
Convergence of DGIB4SL: (a) learning curve of DGIB and (b)–(d): learning curve of each component of the loss of DGIB4SL.

In this section, we analyze the convergence behavior of DGIB4SL. For clarity, we rewrite the DGIB objective function in Eq. 7 as $L^{\text{DGIB}} = L^{\text{CE}} + \beta _{1} L^{\text{KL}} - \beta _{2} L^{\text{DPP}}$, where $L^{\text{CE}}$ is the binary cross-entropy loss, $\beta _{1} L^{KL}$ is the KL-divergence loss, and $-\beta _{2} L^{\text{DPP}}$ represents the DPP loss. Figure 6 illustrates the convergence trends of each component of the DGIB objective. The solid lines correspond to training set values, while the dashed lines represent testing set values. As shown in Fig. 6(a)-(b), both $L^{\text{DGIB}}$ and $L^{\text{CE}}$ experienced a steep decline during the initial epochs, with minimal separation between training and testing curves. This indicates rapid learning and effective generalization by the model. In Fig. 6(c), $L^{\text{KL}}$ shows negligible differences between training and testing curves, suggesting that the compressed input information allows the model to generalize effectively on the test set. In contrast, Fig. 6(d) highlights that $L^{\text{DPP}}$ initially exhibits a more pronounced gap between training and testing curves. However, this gap narrows over time, demonstrating that the model learns diverse representations effectively, albeit at a slower pace compared with other loss components.

#### Parameter sensitivity

**Figure 7:**
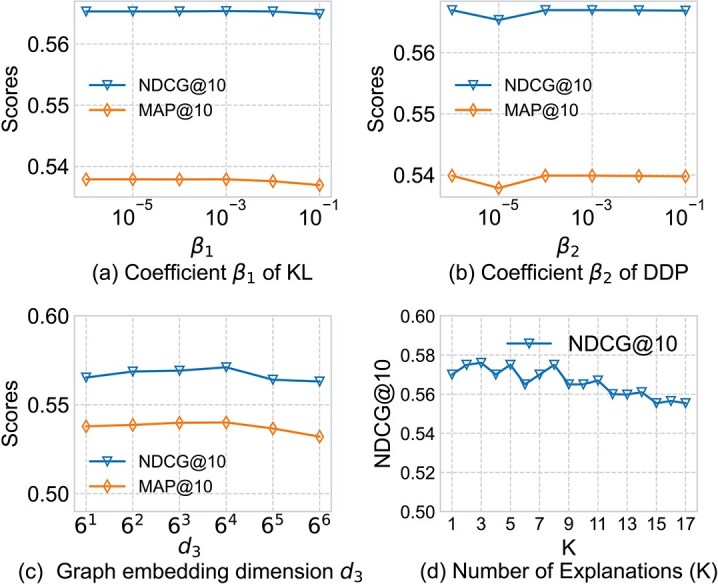
Parameter sensitivity analysis for DGIB4SL on Lagrangian multipliers $\beta _{1}$, $\beta _{2}$, graph embedding dimension $d_{3}$ and the number of explanations $K$ generated for each gene pair on performance.

We explored the impact of the Lagrangian multipliers $\beta _{1}$ and $\beta _{2}$ in Equation 8, the graph representation dimension $d_{3}$ in Equation 9, and the number of explanations $K$ generated by DGIB4SL for each gene pair on SL prediction performance. The performance trend is shown in Fig. 7. From the results, we observed the following: (1) as illustrated in Fig. 7(a)-(b), DGIB4SL’s performance was relatively insensitive to $\beta _{1}$ and $\beta _{2}$. Specifically, $\beta _{1}$ values in the range $[10^{-6}, 10^{-3}]$ and $\beta _{2}$ values in the range $[10^{-4}, 10^{-2}]$ typically yielded robust and reliable performance. For most cases, $\beta _{1} = \beta _{2} = 10^{-4}$ proved to be an optimal choice. (2) Figure 7(c) indicates that increasing $d_{3}$ gradually improved performance, peaking around $d_{3} = 6^{4}$, but began to decline afterward, likely due to overfitting. As the performance gain beyond $d_{3} = 6$ was modest, we opted for $d_{3} = 6$ to simplify the model while maintaining strong performance. (3) Figure 7(d) shows the impact of $K$ within the approximate range of $[1, 17]$ (heuristically estimated; see supplementary materials for details). The results indicate that DGIB4SL performs stably when $K\leq 9$, primarily due to two reasons:

- When the actual number of core subgraphs $c_{i}$ in the $i$th enclosing graph satisfies $c_{i} \geq K$, DGIB4SL only needs to identify at least one core subgraph to make accurate predictions, and the specific number of identified core subgraphs has minimal impact on the results.
- When $c_{i} < K$, the setting of $\beta _{2} = 10^{-4}$ in DGIB biases the trade-off toward relevance (GIB) over diversity (DPP), causing DGIB4SL to prioritize generating core subgraphs relevant to the labels, even if some explanations may overlap.

For $K> 9$, DGIB4SL’s performance declines, primarily due to the increased number of parameters in the relational edge weight module (Eq. S11), which leads to overfitting. We set $K = 3$ for DGIB4SL because most gene pairs have no more than three core subgraphs. This choice helps effectively prevent overfitting and reduce explanation overlap.

### Stability analysis

**Figure 8:**
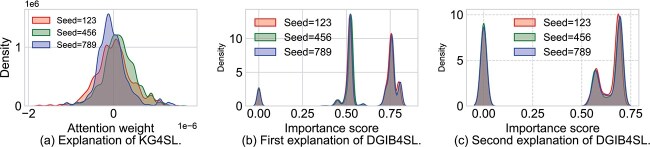
Edge weight distribution of DGIB4SL and KG4SL for the same gene pair (ACTR10, PELO) under different random seeds.

To evaluate the stability of DGIB4SL and attention-based methods, we introduced noise using three distinct random seeds to compare the edge importance distributions. Specifically, we ran DGIB4SL and KG4SL three times with different random seeds. Here, KG4SL was selected as a representative of attention-based methods due to its straightforward design and interpretability. For each run, kernel density estimation [54] was applied to compute the distributions of the importance scores for each edge within the core graph of the gene pair (ACTR10, PELO). As shown in Fig. 8(a), the (unnormalized) attention weight distribution generated by KG4SL is unstable. In contrast, Fig. 8(b) and (c) show that the distributions of edge weight (i.e. $(B_{k}^{uv})_{i,j}$ in Equation 8) generated by DGIB4SL for its two explanations largely overlap across different random seeds, demonstrating the stability of our DGIB4SL’s explainability.

## Conclusion and discussion

We present DGIB4SL, an interpretable SL prediction framework that ensures trustworthy and diverse explanations. DGIB4SL introduces a novel DGIB objective with a Determinantal Point Process constraint to enhance diversity and employs a motif-based strategy to capture high-order graph information. A variational upper bound is proposed to address computational challenges, enabling efficient estimation. Experimental results show that DGIB4SL outperforms all baselines on the SynLethDB 2.0 dataset.

A key limitation of DGIB4SL lies in the fixed number $ K $ for generating explanations, which may result in overlapping or incomplete explanations. Future work could explore an adaptive mechanism to dynamically adjust $ K $ for each enclosing graph. Additionally, DGIB4SL is a general framework for interaction prediction and can be applied to other domains requiring diverse and interpretable explanations, such as drug–drug interaction prediction and functional genomics research.

**Key Points**

- We propose an interpretable knowledge GNN DGIB4SL that predicts SL interactions with diverse explanations.
- We use the GIB principle to define a core subgraph of a gene pair, and extend the GIB objective to handle data with multiple core subgraphs, resulting in DGIB, which serves as the objective for DGIB4SL.
- We apply motif-based GNNs to capture high-order graph structures.
- The model’s effectiveness is validated through real-world data and case studies.

## Supplementary Material

DGIB4SL_suppv4_bbaf142

supplementary_materials_final_bbaf142(1)

## Data Availability

Our source codes and pre-processed datasets are publicly available via https://github.com/CXX1113/DGIB4SL.
