# Supplementary material for: Interpretable high-order knowledge graph neural network for predicting synthetic lethality in human cancers
Source: Brief Bioinform. 2025 Apr 7;26(2):bbaf142. doi: 10.1093/bib/bbaf142 (PMC11975366; doi:10.1093/bib/bbaf142)
Supplement: DGIB4SL_suppv4_bbaf142 [file dgib4sl_suppv4_bbaf142.pdf]

Journal Title Here, 2022, pp. 1–6

doi: DOI HERE
Advance Access Publication Date: Day Month Year
Paper

PAPER

Supplementary Materials of DGIB4SL

Xuexin Chen,[1] Ruichu Cai,[1,2,*] Zhengting Huang,[1] Zijian Li,[3] Jie Zheng[4,5] and Min Wu[6,*]

[1]School of Computer Science, Guangdong University of Technology, 510006, Guangdong, China, [2]Pazhou Laboratory (Huangpu), Guangzhou, China, [3]Mohamed bin Zayed University of Artificial Intelligence, Abu Dhabi, [4]School of Information Science and Technology, ShanghaiTech University, 201210, China, [5]Shanghai Engineering Research Center of Intelligent Vision and Imaging, ShanghaiTech University, 201210, China and [6]Institute for Infocomm Research (I^{2}R), A*STAR, 178902, Singapore

*Corresponding authors. cairuichu@gmail.com ,wumin@i2r.a-star.edu.sg

FOR PUBLISHER ONLY Received on Date Month Year; revised on Date Month Year; accepted on Date Month Year

Abstract

Key words: synthetic lethality, machine learning explainability, graph neural network, information bottleneck

Explainability in graph neural networks

As GNNs are increasingly applied to SL prediction, understanding the reasoning behind their predictions becomes critical. The explainability of GNNs can be broadly categorized into two classes: post-hoc explanations and self-explainable GNNs. Post-hoc methods build an additional explainer to interpret a trained GNN, using techniques such as gradients [1], perturbation [2, 3], or interpretable linear agents [4]. However, these methods often fail to reveal the true reasoning process due to the inherent non-convexity and complexity of GNNs [5, 6]. Self-explainable GNNs address the limitations of post-hoc approaches by providing predictions and explanations simultaneously. Two major directions in this area are: (1) Information bottleneck (IB) approaches: these methods use the IB principle [7] as a training objective to extract subgraphs closely related to graph labels [8, 9, 10, 11]. (2) Prototype learning: approaches like ProtGNN [12] identify subgraphs most relevant to graph patterns associated with specific classes. Beyond these, other methods like DIR [13] identifies causal patterns via distribution interventions and models classifiers based on causal and non-causal components, while GREA [14] introduces environment replacement to generate virtual data examples for better pattern identification. Despite their advantages, these methods face challenges when applied to SL prediction. Similar to KG-based approaches, self-explainable GNNs are limited in generating multiple explanations and capturing higher-order graph structures essential for prediction.

Intuition for Measuring Diversity in Eq. 3

Intuitively, each entry in the matrix L^{uv} in Eq. 4 represents the similarity between the representations of two elements in the set, computed using dot products. Eq. 3 measures the diversity of elements within a set by leveraging the principle that, when the feature vectors of the elements are not linearly correlated, the volume of the hypercube they form is maximized. Consequently, the determinant of L^{uv} in Eq. 3 quantifies this volume, effectively capturing the diversity of the feature vectors [15].

Proof of Eq. 6

Proposition 1 *(Upper bound of $-I(Y;\widetilde{G}_k^{uv})$). For a graph G^{uv} with label Y and the k-th IB-Graph $\widetilde{G}_k^{uv}$ learned from G^{uv}, we have*

$$-I\left(Y;\widetilde{G}_k^{uv}\right) \le -\mathbb{E}_{Y,\widetilde{G}_k^{uv}}\left[\log q_\theta(Y|\widetilde{G}_k^{uv})\right] + H(Y), \tag{S1}$$

where $q_\theta(Y|\widetilde{G}_k^{uv})$ parameterized by θ is the variational approximation of $p(Y|\widetilde{G}_k^{uv})$.

Proof

$$\begin{aligned} I(Y;\widetilde{G}_k^{uv}) &= \int\int p(Y,\widetilde{G}_k^{uv})\log\frac{p(Y,\widetilde{G}_k^{uv})}{p(Y)p(\widetilde{G}_k^{uv})}dY d\widetilde{G}_k^{uv} \\ &= \int\int p(Y,\widetilde{G}_k^{uv})\log\frac{p(Y|\widetilde{G}_k^{uv})}{p(Y)}dY d\widetilde{G}_k^{uv} \\ &= \mathbb{E}_{Y,\widetilde{G}_k^{uv}}\left[\log\frac{p(Y|\widetilde{G}_k^{uv})}{p(Y)}\right] \end{aligned} \tag{S2}$$

Since $p(Y|\widetilde{G}_k^{uv})$ is intractable, we introduce a variational approximation $q_\theta(Y|\widetilde{G}_k^{uv})$ for it. Then we have

$$\begin{aligned} &I(Y;\widetilde{G}_k^{uv}) \\ &= \mathbb{E}_{Y,\widetilde{G}_k^{uv}}\left[\log\frac{q_\theta(Y|\widetilde{G}_k^{uv})}{p(Y)}\frac{p(Y|\widetilde{G}^{(k)})}{q_\theta(Y|\widetilde{G}_k^{uv})}\right] \\ &= \mathbb{E}_{Y,\widetilde{G}_k^{uv}}\left[\log\frac{q_\theta(Y|\widetilde{G}_k^{uv})}{p(Y)}\right] + \mathbb{E}_{Y,\widetilde{G}_k^{uv}}\left[\log\frac{p(Y|\widetilde{G}_k^{uv})}{q_\theta(Y|\widetilde{G}_k^{uv})}\right], \end{aligned} \tag{S3}$$

© The Author 2022. Published by Oxford University Press. All rights reserved. For permissions, please e-mail: journals.permissions@oup.com

Table S1. Notations and Descriptions.

Notations	Descriptions
$(T)_{ij}$, $(T)_i$	Element at the i-th row and the j-th column of matrix T, and the i-the row of T.
G=(A, X, E)	Joint graph data of the SL graph and KG.
A, X, E	Adjacency matrix, node feature matrix, and edge feature matrix of G.
G^{uv}=(A^{uv}, X^{uv}, E^{uv})	Enclosing graph data for genes u and v.
Y	Binary SL interaction label of the gene pair (u, v).
$K \geq 1$	Number of explanations generated by DGIB4SL for a gene pair.
d_0, d_1, d_2, d_3	Dimensions of input features for nodes and relationships, relational space, and graph representation.
$\widetilde{G}_k^{uv}$=$(\widetilde{A}_k^{uv}, \widetilde{X}_k^{uv})$	k-th IB-graph data of G^{uv}.
$\widetilde{Z}_k^{uv} \in \mathbb{R}^{d_3}$	Graph representation of $\widetilde{G}_k^{uv}$.
$f_\phi^\mu(\widetilde{Z}_k^{uv})$, $f_\phi^\Sigma(\widetilde{Z}_k^{uv})$	Mean vector and diagonal covariance matrix of the distribution of $\widetilde{Z}_k^{uv}$.
$L^{uv} \in \mathbb{R}^{K\times K}$	Inner product of K graph representations $\widetilde{Z}_1^{uv}$, ..., $\widetilde{Z}_K^{uv}$.
B_k^{uv}	Learnable edge importance weight matrix of $\widetilde{G}_k^{uv}$.
β_1, β_2	Coefficients of KL and DPP in the DGIB objective function.
Ω_i, M_i^{uv}	Motif-based adjacency matrix of $\widetilde{G}^{uv}$ for a given motif Ω_i.
W_k^1, W_k^2, θ, ϕ	Learnable parameters.

where

$$\begin{aligned}
&\mathbb{E}_{Y,\widetilde{G}_k^{uv}}\left[\log\frac{p(Y|\widetilde{G}_k^{uv})}{q_\theta(Y|\widetilde{G}_k^{uv})}\right]\\
&=\int\int p(\widetilde{G}_k^{uv})p(Y|\widetilde{G}_k^{uv})\log\frac{p(Y|\widetilde{G}_k^{uv})}{q_\theta(Y|\widetilde{G}_k^{uv})}dY d\widetilde{G}_k^{uv}\\
&=\mathbb{E}_{\widetilde{G}_k^{uv}}[D_{\mathrm{KL}}(p(Y|\widetilde{G}_k^{uv})||q_\theta(Y|\widetilde{G}_k^{uv}))].
\end{aligned}\tag{S4}$$

Plug Eq. S4 into Eq. S3, we have

$$\begin{aligned}
&I(Y;\widetilde{G}_k^{uv})\\
&=\mathbb{E}_{Y,\widetilde{G}_k^{uv}}\left[\log\frac{q_\theta(Y|\widetilde{G}_k^{uv})}{p(Y)}\right]+\mathbb{E}_{\widetilde{G}_k^{uv}}[D_{\mathrm{KL}}(p(Y|\widetilde{G}_k^{uv})||q_\theta(Y|\widetilde{G}_k^{uv}))]\\
&\geq\mathbb{E}_{Y,\widetilde{G}_k^{uv}}\left[\log\frac{q_\theta(Y|\widetilde{G}_k^{uv})}{p(Y)}\right]\text{ (non-negativity of KL Divergence)}\\
&=\mathbb{E}_{Y,\widetilde{G}_k^{uv}}\left[\log q_\theta(Y|\widetilde{G}_k^{uv})\right]-\mathbb{E}_{Y,\widetilde{G}_k^{uv}}[\log p(Y)]\\
&=\mathbb{E}_{Y,\widetilde{G}_k^{uv}}\left[\log q_\theta(Y|\widetilde{G}_k^{uv})\right]-H(Y)\text{ (normalization of}\\
&\qquad\text{PDF } p(\widetilde{G}_k^{uv}|Y)\\
&\Rightarrow -I(Y;\widetilde{G}_k^{uv})\leq-\mathbb{E}_{Y,\widetilde{G}_k^{uv}}\left[\log q_\theta(Y|\widetilde{G}_k^{uv})\right]+H(Y)
\end{aligned}\tag{S5}$$

where $H(Y)$ is the entropy of label Y, which can be ignored in the optimization procedure. □

Proposition 2 *(Upper bound of $I(G^{uv};\widetilde{G}_k^{uv})$). For a graph G^{uv} and the k-th IB-Graph $\widetilde{G}_k^{uv}$ learned from G^{uv}, we have*

$$I(G^{uv};\widetilde{G}_k^{uv})\leq\mathbb{E}_{G^{uv}}[D_{KL}(q_\phi(\widetilde{G}_k^{uv}|G^{uv})||q(\widetilde{G}_k^{uv})],\tag{S6}$$

where $q(\widetilde{G}_k^{uv})$=$\sum_{G^{uv}}p(G^{uv})q_\phi(\widetilde{G}_k^{uv}|G^{uv})$ and $q_\phi(\widetilde{G}_k^{uv}|G^{uv})$ parameterized by ϕ is the variational approximation of $p(\widetilde{G}_k^{uv}|G^{uv})$, $D_{KL}(\cdot)$ denotes the Kullback-Leibler (KL) divergence.

Proof

$$\begin{aligned}
I(G^{uv};\widetilde{G}_k^{uv})&=\int\int p(G^{uv},\widetilde{G}_k^{uv})\log\frac{p(G^{uv},\widetilde{G}_k^{uv})}{p(G^{uv})p(\widetilde{G}_k^{uv})}dG^{uv}d\widetilde{G}_k^{uv}\\
&=\int\int p(G^{uv},\widetilde{G}_k^{uv})\log\frac{q_\phi(\widetilde{G}_k^{uv}|G^{uv})}{p(\widetilde{G}_k^{uv})}dG^{uv}d\widetilde{G}_k^{uv}\\
&=\mathbb{E}_{G^{uv},\widetilde{G}_k^{uv}}\left[\log\frac{q_\phi(\widetilde{G}_k^{uv}|G^{uv})}{p(\widetilde{G}_k^{uv})}\right].
\end{aligned}\tag{S7}$$

Since $p(\widetilde{G}_k^{uv})$ is intractable, we introduce a variational approximation $q(\widetilde{G}_k^{uv})$=$\sum_{G^{uv}}p(G^{uv})q_\phi(\widetilde{G}_k^{uv}|G^{uv})$ for the marginal distribution $p(\widetilde{G}_k^{uv})$. Then we have

$$\begin{aligned}
I(G^{uv};\widetilde{G}_k^{uv})&=\mathbb{E}_{G^{uv},\widetilde{G}_k^{uv}}\left[\log\frac{q(\widetilde{G}_k^{uv})}{p(\widetilde{G}_k^{uv})}\frac{q_\phi(\widetilde{G}_k^{uv}|G^{uv})}{q(\widetilde{G}_k^{uv})}\right]\\
&=\mathbb{E}_{G^{uv},\widetilde{G}_k^{uv}}\left[\log\frac{q_\phi(\widetilde{G}_k^{uv}|G^{uv})}{q(\widetilde{G}_k^{uv})}\right]+\mathbb{E}_{G^{uv},\widetilde{G}_k^{uv}}\left[\log\frac{q(\widetilde{G}_k^{uv})}{p(\widetilde{G}_k^{uv})}\right]\\
&=\mathbb{E}_{G^{uv},\widetilde{G}_k^{uv}}\left[\log\frac{q_\phi(\widetilde{G}_k^{uv}|G^{uv})}{q(\widetilde{G}_k^{uv})}\right]-\mathbb{E}_{G^{uv},\widetilde{G}_k^{uv}}\left[\log\frac{p(\widetilde{G}_k^{uv})}{q(\widetilde{G}_k^{uv})}\right],
\end{aligned}\tag{S8}$$

where

$$\begin{aligned}
&\mathbb{E}_{G^{uv},\widetilde{G}_k^{uv}}\left[\log\frac{p(\widetilde{G}_k^{uv})}{q(\widetilde{G}_k^{uv})}\right]\\
&=\int p(\widetilde{G}_k^{uv})\log\frac{p(\widetilde{G}_k^{uv})}{q(\widetilde{G}_k^{uv})}\int p(G^{uv}|\widetilde{G}_k^{uv})dG^{uv}d\widetilde{G}_k^{uv}\\
&=\int p(\widetilde{G}_k^{uv})\log\frac{p(\widetilde{G}_k^{uv})}{q(\widetilde{G}_k^{uv})}d\widetilde{G}_k^{uv}\\
&=D_{\mathrm{KL}}(p(\widetilde{G}_k^{uv})||q(\widetilde{G}_k^{uv}))
\end{aligned}\tag{S9}$$

Plug Eq. S9 into Eq. S8, we have

$$\begin{aligned}
&I(G^{uv};\widetilde{G}_k^{uv})\\
&=\mathbb{E}_{G^{uv},\widetilde{G}_k^{uv}}\left[\log\frac{q_\phi(\widetilde{G}_k^{uv}|G^{uv})}{q(\widetilde{G}_k^{uv})}\right]-D_{\mathrm{KL}}(p(\widetilde{G}_k^{uv})||q(\widetilde{G}_k^{uv}))\\
&\leq\mathbb{E}_{G^{uv},\widetilde{G}_k^{uv}}\left[\log\frac{q_\phi(\widetilde{G}_k^{uv}|G^{uv})}{q(\widetilde{G}_k^{uv})}\right]\text{ (non-negativity of KL Divergence)}\\
&=\mathbb{E}_{G^{uv}}[D_{\mathrm{KL}}(q_\phi(\widetilde{G}_k^{uv}|G^{uv})||q(\widetilde{G}_k^{uv})]
\end{aligned}\tag{S10}$$

□

The calculation method of B_k^{uv}

The calculation of B_k^{uv} is as follows: For any edge (i,j) not present in the enclosing graph G^{uv}, $(B_k^{uv})_{i,j}$ is fixed to 0, since our goal is to extract a subgraph from G^{uv}. For edges (i,j) in G^{uv}=(A^{uv}, X^{uv}, E^{uv}), inspired by the approaches of Wang et al. [16], we optimize $(B_k^{uv})_{i,j}$ jointly with relational graph learning by the following function (corresponding to ψ_k in Fig. 2):

$$(B_k^{uv})_{i,j} := (W_k^1(X^{uv})_j) \cdot \tanh(W_k^1(X^{uv})_i + W_k^2(E^{uv})_r)^{\mathrm{T}}, \tag{S11}$$

where $W_k^1 \in \mathbb{R}^{d_2 \times d_0}$ and $W_k^2 \in \mathbb{R}^{d_2 \times d_1}$ are learnable weight matrices for mapping node and relation features into the same space, respectively. Here, $(E^{uv})_r \in \mathbb{R}^{d_1}$ represents the feature of relation r in the triple (i,r,j) and $\tanh(\cdot)$ is a nonlinear activation function. This formulation ensures that $(B_k^{uv})_{i,j}$ depends on the distance between the features $(X^{uv})_i$, $(X^{uv})_j$ of nodes i and j in the space defined by relation r [16]. Intuitively, $(B_k^{uv})_{i,j}$ reflects the SL prediction-specific importance of the edge, with smaller values indicating noise that should be assigned lower weights or excluded. Note that $\widetilde{A}_k^{uv}$ is not differentiable with respect to B_k^{uv} due to Bernoulli sampling, we employ the concrete relaxation [17] to approximate sampling for the IB-Graph:

$$(\widetilde{A}_k^{uv})_{i,j} = \mathrm{sigmoid}(\frac{1}{\tau}(\log \frac{(B_k^{uv})_{i,j}}{1-(B_k^{uv})_{i,j}} + \log \frac{\epsilon}{1-\epsilon})), \tag{S12}$$

where $\epsilon \sim \mathrm{Uniform}(0,1)$ and $\tau \in \mathbb{R}^+$ is the temperature of the concrete distribution.

Construction of the IB-Graph

The construction process of the IB-Graph, as shown in Fig. S1, involves four steps, which slightly differ between the training and testing phases. During the training phase:

1. **Input**: The enclosing graph data $G = (A, X, E)$ (with the superscript uv omitted for simplicity) is provided for a given gene pair (u, v).
2. **Edge weight estimation**: The weights of all edges in G are estimated using Eq. S11, which serves as the edge-weighting module.
3. **Subgraph extraction**: These edge weights are treated as parameters of independent Bernoulli variables, and random sampling assigns each edge a value of 1 or 0.
4. **Subgraph output**: The sampled edge values are aggregated to form a binary adjacency matrix $\tilde{A}_k$, representing the adjacency matrix of the k-th IB-graph. The node feature matrix $\tilde{X}_k$ is retained as equal to X, as our focus is on edge-level explanation.

Note that sampling from a Bernoulli distribution is not differentiable, the Gumbel-Softmax trick is thus used during optimization, as detailed in Eq. S12.

In the testing phase, the process differs only in the third step. Instead of sampling, a predefined importance threshold ϵ_k, derived from the learned importance distribution, determines whether an edge exists. In our experiments, this threshold is set as the median of the ranked edge weights in B_k. The reason why the subgraph extraction methods in the third step differ is that subgraph selection during training is intended to optimize the model's parameters, while during testing, it aims to infer the best estimation of the explanatory subgraph. This difference also aligns with the standard practice in GIB-based methods, as seen in the literature [8, 10].

Detailed information of baselines

Table S2. Numbers of the entities in the SynLethKG.

Type	No. of entities
Gene	25,260
Biological process	12,703
Side effect	5,702
Molecular function	3,203
Pathway	2,069
Compound	2,065
Cellular component	1,670
Symptom	427
Anatomy	400
Pharmacologic class	377
Disease	136

Table S3. Summary of the relationship in the SynLethKG.

Type	No. of entities
(Gene, Regulates,Gene)	267,791
(Gene, Interacts, Gene)	148,379
(Gene, Covaries, Gene)	62,987
(Anatomy, Expresses, Gene)	617,175
(Disease, Associates, Gene)	24,328
(Disease, Upregulates, Gene)	7,730
(Compound, Downregulates, Gene)	21,526
(Disease, Downregulates, Gene)	7,616
(Compound, Binds, Gene)	16,323
(Compound, Upregulates, Gene)	19,200
(Anatomy, Upregulates, Gene)	26
(Anatomy, Downregulates, Gene)	31
(Gene, Participates, Cellular component)	97,652
(Gene, Participates, Biological process)	619,712
(Compound, Causes, SideEffect)	139,428
(Gene, Participates, Molecular function)	110,042
(Gene, Participates, Pathway)	57,441
(Compound, Treats, Disease)	752
(Compound, Resembles, Compound)	6,266
(Pharmacologic Class, Includes, compound)	1,205
(Disease, Localizes, Anatomy)	3,373
(Disease, Presents, Symptom)	3,401
(Compound, Palliates, Disease)	384
(Disease, Resembles, Disease)	404

Table S4. Summary of the relationship in the core graphs in Fig. 4 output by DGIB4SL.

Label	Description
includes_PCiC	Pharmacological class includes compound.
palliates_Cpd	Compound palliates disease.
participates_GpMF	Gene participates in molecular function.
presents_Dps	Disease presents symptom.

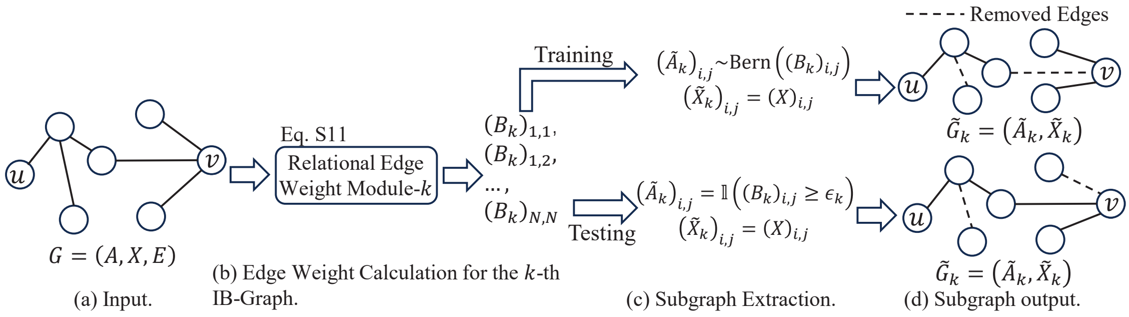

Fig. S1. Overview for extracting the k-th IB-graph from the enclosing graph $G{=}(A, X, E)$ (for simplicity, the superscript uv is omitted) around the pair of genes u and v.

We evaluated our DGIB4SL against two categories of methods: matrix factorization (MF)-based methods and graph neural network (GNN)-based methods, selecting thirteen recent approaches.

- **GRSMF** [18] reconstructs the SL interaction graph using graph-regularized self-representative MF, incorporating PPI and GO for regularization.
- **SL^{2}MF** [19] employs logistic MF for SL prediction, integrating importance weighting and PPI/GO information.
- **CMFW** [20] combines multiple data sources using collective matrix factorization to generate latent representations.

GNN-based methods include:

- **DDGCN** [21] utilizes GCN with SL interaction matrix features and applies dropout techniques to address sparse graphs.
- **GCATSL** [22] uses a dual attention mechanism with SL, PPI, and GO as input graphs to complete the SL graph.
- **SLMGAE** [23] implements a multi-view graph autoencoder, integrating SL, PPI, and GO graphs for prediction.
- **MGE4SL** [24] leverages a Multi-Graph Ensemble to combine PPI, GO, and Pathway data using neural network embeddings.
- **PTGNN** [25] pre-trains GNNs with various data sources and graph-based reconstruction features.
- **KG4SL** [26] is the first GNN-based model to integrate a knowledge graph for SL prediction, utilizing an attention mechanism.
- **PiLSL** [27] extracts pairwise local subgraphs for SL prediction and integrates multi-omics data with attention mechanisms.
- **NSF4SL** [28] uses contrastive learning with pre-trained KG-based features (e.g., TransE[29]) as input for neural embeddings.
- **KR4SL** [30] encodes structural information, textual semantic information, and sequential semantics for gene representations, and uses different attention mechanisms to select important edges in each hop.
- **SLGNN** [31] generates gene embeddings with factor-based message passing and identifies important factors through attention mechanisms, where factors consist of relationships in the KG.

Explainability evaluation metrics

Given the lack of ground-truth explanations in the SL dataset, we employed two metrics to evaluate explanation **accuracy**:

- **Infidelity** [32] measures explanation faithfulness, where more important features (e.g., edges or relational features) should cause larger prediction changes when altered. It introduces random perturbations to the input features, weights them by importance, and compares the changes in features and predictions to evaluate consistency.
- **Sparseness** [33] evaluates explanation sparsity using the Gini index. Higher Sparseness indicates that the explanation concentrates on fewer important features, effectively reducing redundant information.

Implementation details

To evaluate the effectiveness of our DGIB4SL, we adopted the same data processing, splitting, and evaluation metrics as SLB [34]. For dataset processing, the number of positive samples (i.e. known SL pairs) was balanced with an equal number of negative samples. Negative samples were generated using a common strategy of randomly selecting gene pairs from unknown samples.

The configuration of DGIB4SL and baseline models for the SL prediction task was as follows: For our DGIB4SL, we set $d_2{=}d_3{=}6$ (Eq. S11, Eq. 4). Next, the coefficients β_1 and β_2 in Eq. 6 were set $\beta_1{=}\beta_2{=}10^{-4}$. We further set the number of explanations K in Eq. 6 for each sample to 3 (for the appropriate range of K, please refer to the next section). We used the Stochastic Gradient Descent (SGD) optimizer with a learning rate 0.005. The maximum number of training epochs was set as 5. For the baselines, we tuned their settings according to SLB [34]. Specifically, the output embedding dimension was set to 256 and the hidden embedding dimension was set to 512. The learning rate was tuned empirically. An early stopping strategy was used to avoid overfitting during training. The number of layers in most GNNs was set to 1, except for KR4SL, which used a 3-layer GNN to generate path-based explanations. We set the rank position C in the ranking metrics to 10 and 50 to represent 0.1% and 0.7% of the 7,183 candidate gene pairs, offering two levels of ranking difficulty. These values also align with those used in related studies (e.g., NSF4SL [28], KR4SL [30]).

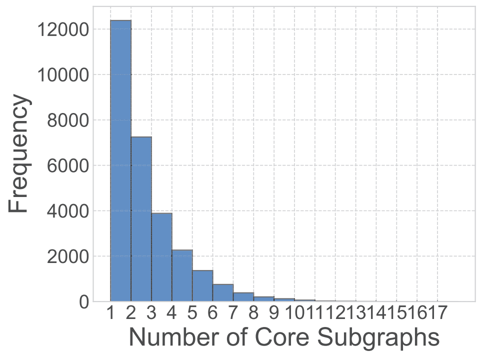

Fig. S2. Frequency histogram of estimated core subgraphs for SL pairs enclosing graphs in the training set.

Determining the range of K

To determine the range of K (the number of explanations generated by DGIB4SL for each gene pair), it is necessary to clearly specify its lower and upper bounds. The lower bound is the minimum number of core subgraphs across all SL gene pairs, which is clearly 1. The upper bound represents the maximum number of core subgraphs among all possible SL gene pairs. Since calculating the exact number of core subgraphs for all SL gene pairs is unrealistic, we use a heuristic approach to estimate the range of K based on the training set. This method is guided by the intuition that core subgraphs are densely connected internally and sparsely connected externally, which is divided into 4 steps:

1. For each SL gene pair in the training set, we compute node importance scores in the enclosing graph using the PageRank (PR) algorithm [35].
2. Nodes with importance scores below the 90-*th* percentile are considered unimportant and removed from the graph.
3. The number of connected components formed by the remaining nodes serves as an estimate of the number of core subgraphs in the given enclosing graph.
4. Fig. S2 presents the frequency histogram of core subgraph counts in the training set. Thus, the approximate range for K is [1, 17].

References

1. Mukund Sundararajan, Ankur Taly, and Qiqi Yan. Axiomatic attribution for deep networks. In *International conference on machine learning*, pages 3319–3328. PMLR, 2017.
2. Zhitao Ying, Dylan Bourgeois, Jiaxuan You, Marinka Zitnik, and Jure Leskovec. Gnnexplainer: Generating explanations for graph neural networks. *Advances in neural information processing systems*, 32, 2019.
3. Dongsheng Luo, Wei Cheng, Dongkuan Xu, Wenchao Yu, Bo Zong, Haifeng Chen, and Xiang Zhang. Parameterized explainer for graph neural network. *NeurIPS 2020*.
4. Qiang Huang, Makoto Yamada, Yuan Tian, Dinesh Singh, and Yi Chang. Graphlime: Local interpretable model explanations for graph neural networks. *IEEE Trans. Knowl. Data Eng.*, 35(7):6968–6972, 2023.
5. Jingyu Peng, Qi Liu, Linan Yue, Zaixi Zhang, Kai Zhang, and Yunhao Sha. Towards few-shot self-explaining graph neural networks. volume 14946 of *Lecture Notes in Computer Science*, pages 109–126. Springer, 2024.
6. Antonio Longa, Steve Azzolin, Gabriele Santin, Giulia Cencetti, Pietro Liò, Bruno Lepri, and Andrea Passerini. Explaining the explainers in graph neural networks: a comparative study. *ACM Computing Surveys*, 2024.
7. Naftali Tishby, Fernando C. N. Pereira, and William Bialek. The information bottleneck method. *CoRR*, physics/0004057, 2000.
8. Siqi Miao, Mia Liu, and Pan Li. Interpretable and generalizable graph learning via stochastic attention mechanism. In *ICML 2022*.
9. Qingyun Sun, Jianxin Li, Hao Peng, Jia Wu, Xingcheng Fu, Cheng Ji, and S Yu Philip. Graph structure learning with variational information bottleneck. In *AAAI 2022*.
10. Junchi Yu, Jie Cao, and Ran He. Improving subgraph recognition with variational graph information bottleneck. In *Proceedings of the IEEE/CVF Conference on Computer Vision and Pattern Recognition*, pages 19396–19405, 2022.
11. Sangwoo Seo, Sungwon Kim, and Chanyoung Park. Interpretable prototype-based graph information bottleneck. *NeurIPS 2024*.
12. Zaixi Zhang, Qi Liu, Hao Wang, Chengqiang Lu, and Cheekong Lee. Protgnn: Towards self-explaining graph neural networks. In *Proceedings of the AAAI Conference on Artificial Intelligence*, volume 36, pages 9127–9135, 2022.
13. Yingxin Wu, Xiang Wang, An Zhang, Xiangnan He, and Tat-Seng Chua. Discovering invariant rationales for graph neural networks. In *The Tenth International Conference on Learning Representations, ICLR 2022, Virtual Event, April 25-29, 2022*, 2022.
14. Gang Liu, Tong Zhao, Jiaxin Xu, Tengfei Luo, and Meng Jiang. Graph rationalization with environment-based augmentations. In *Proceedings of the 28th ACM SIGKDD Conference on Knowledge Discovery and Data Mining*, pages 1069–1078, 2022.
15. Alex Kulesza, Ben Taskar, et al. Determinantal point processes for machine learning. *Foundations and Trends® in Machine Learning*, 5(2–3):123–286, 2012.
16. Xiang Wang, Xiangnan He, Yixin Cao, Meng Liu, and Tat-Seng Chua. Kgat: Knowledge graph attention network for recommendation. In *KDD*, 2019.
17. Eric Jang, Shixiang Gu, and Ben Poole. Categorical reparameterization with gumbel-softmax. In *ICLR 2017*.
18. Jiang Huang, Min Wu, Fan Lu, Le Ou-Yang, and Zexuan Zhu. Predicting synthetic lethal interactions in human cancers using graph regularized self-representative matrix factorization. *BMC Bioinform.*, 20-S(19):657, 2019.
19. Yong Liu, Min Wu, Chenghao Liu, Xiaoli Li, and Jie Zheng. Sl^2mf: Predicting synthetic lethality in human cancers via logistic matrix factorization. *IEEE ACM Trans. Comput. Biol. Bioinform.*, 17(3):748–757, 2020.
20. Herty Liany, Anand Jeyasekharan, and Vaibhav Rajan. Predicting synthetic lethal interactions using heterogeneous data sources. *Bioinform.*, 36(7):2209–2216, 2020.
21. Ruichu Cai, Xuexin Chen, Yuan Fang, Min Wu, Yuexing Hao, and Jonathan D. Wren. Dual-dropout graph convolutional network for predicting synthetic lethality in human cancers. *Bioinform.*, 36(16):4458–4465, 2020.
22. Yahui Long, Min Wu, Yong Liu, Jie Zheng, Chee Keong Kwoh, Jiawei Luo, and Xiaoli Li. Graph contextualized attention network for predicting synthetic lethality in human cancers. *Bioinform.*, 37(16):2432–2440, 2021.

23. Zhifeng Hao, Di Wu, Yuan Fang, Min Wu, Ruichu Cai, and Xiaoli Li. Prediction of synthetic lethal interactions in human cancers using multi-view graph auto-encoder. *IEEE J. Biomed. Health Informatics*, 25(10):4041–4051, 2021.
24. Mincai Lai, Guangyao Chen, Haochen Yang, Jingkang Yang, Zhihao Jiang, Min Wu, and Jie Zheng. Predicting synthetic lethality in human cancers via multi-graph ensemble neural network. In *43rd Annual International Conference of the IEEE Engineering in Medicine & Biology Society*. IEEE, 2021.
25. Yahui Long, Min Wu, Yong Liu, Yuan Fang, Chee Keong Kwoh, Jinmiao Chen, Jiawei Luo, and Xiaoli Li. Pre-training graph neural networks for link prediction in biomedical networks. *Bioinform.*, 38(8):2254–2262, 2022.
26. Shike Wang, Fan Xu, Yunyang Li, Jie Wang, Ke Zhang, Yong Liu, Min Wu, and Jie Zheng. Kg4sl: knowledge graph neural network for synthetic lethality prediction in human cancers. *Bioinformatics*, 37, 2021.
27. Xin Liu, Jiale Yu, Siyu Tao, Beiyuan Yang, Shike Wang, Lin Wang, Fang Bai, and Jie Zheng. Pilsl: pairwise interaction learning-based graph neural network for synthetic lethality prediction in human cancers. *Bioinformatics*, 38(Supplement_2):ii106–ii112, 2022.
28. Shike Wang, Yimiao Feng, Xin Liu, Yong Liu, Min Wu, and Jie Zheng. Nsf4sl: negative-sample-free contrastive learning for ranking synthetic lethal partner genes in human cancers. *Bioinformatics*, 38(Supplement_2):ii13–ii19, 2022.
29. Antoine Bordes, Nicolas Usunier, Alberto García-Durán, Jason Weston, and Oksana Yakhnenko. Translating embeddings for modeling multi-relational data. In *Advances in 27th Annual Conference on Neural Information Processing Systems 2013.*
30. Ke Zhang, Min Wu, Yong Liu, Yimiao Feng, and Jie Zheng. Kr4sl: knowledge graph reasoning for explainable prediction of synthetic lethality. *Bioinformatics*, 39, 2023.
31. Yan Zhu, Yuhuan Zhou, Yang Liu, Xuan Wang, and Junyi Li. Slgnn: synthetic lethality prediction in human cancers based on factor-aware knowledge graph neural network. *Bioinformatics*, 39(2):btad015, 2023.
32. Chih-Kuan Yeh, Cheng-Yu Hsieh, Arun Suggala, David I Inouye, and Pradeep K Ravikumar. On the (in) fidelity and sensitivity of explanations. *Advances in neural information processing systems*, 32, 2019.
33. Prasad Chalasani, Jiefeng Chen, Amrita Roy Chowdhury, Xi Wu, and Somesh Jha. Concise explanations of neural networks using adversarial training. In *International Conference on Machine Learning*, pages 1383–1391. PMLR, 2020.
34. Yimiao Feng, Yahui Long, He Wang, Yang Ouyang, Quan Li, Min Wu, and Jie Zheng. Benchmarking machine learning methods for synthetic lethality prediction in cancer. *Nature Communications*, 15:9058, 2024.
35. Lawrence Page, Sergey Brin, Rajeev Motwani, and Terry Winograd. The pagerank citation ranking: Bringing order to the web. Technical Report 1999-66, Stanford InfoLab, November 1999.
